# Effects of an *Agaricus blazei* Aqueous Extract Pretreatment on Paracetamol-Induced Brain and Liver Injury in Rats

**DOI:** 10.1155/2013/469180

**Published:** 2013-07-25

**Authors:** Andréia A. Soares, Andrea L. de Oliveira, Anacharis B. Sá-Nakanishi, Jurandir F. Comar, Ana P. S. Rampazzo, Fernando A. Vicentini, Maria R. M. Natali, Sandra M. Gomes da Costa, Adelar Bracht, Rosane M. Peralta

**Affiliations:** Department of Biochemistry, University of Maringá, 87020900 Maringá, PR, Brazil

## Abstract

The action of an *Agaricus blazei* aqueous extract pretreatment on paracetamol injury in rats was examined not only in terms of the classical indicators (e.g., levels of hepatic enzymes in the plasma) but also in terms of functional and metabolic parameters (e.g., gluconeogenesis). Considering solely the classical indicators for tissue damage, the results can be regarded as an indication that the *A. blazei* extract is able to provide a reasonable degree of protection against the paracetamol injury in both the hepatic and brain tissues. The *A. blazei* pretreatment largely prevented the increased levels of hepatic enzymes in the plasma (ASP, ALT, LDH, and ALP) and practically normalized the TBARS levels in both liver and brain tissues. With respect to the functional and metabolic parameters of the liver, however, the extract provided little or no protection. This includes morphological signs of inflammation and the especially important functional parameter gluconeogenesis, which was impaired by paracetamol. Considering these results and the long list of extracts and substances that are said to have hepatoprotective effects, it would be useful to incorporate evaluations of functional parameters into the experimental protocols of studies aiming to attribute or refute effective hepatoprotective actions to natural products.

## 1. Introduction

The mushrooms have generally been considered functional foods and, consequently, important sources of bioactive compounds, with the *β*-glucans, terpenes, phenolics, steroids, and nucleosides being so far the most important. These compounds are said to exert several effects, the most prominent being the antitumoral, antimutagenic, anticytotoxic, antimicrobial, antioxidant, hepatoprotective, anti-inflammatory, and hypoglycemic and antidislipidemic actions [1, 2]. *Agaricus blazei *Murrill is a basidiomycete whose popular name is “sun mushroom” and which has become the subject of great interest, due to its nutritional value and pharmacological properties [3]. In Brazil *A. blazei* is greatly consumed in the form of concentrated extracts or teas and popularly used against a variety of diseases such as diabetes, atherosclerosis, hypercholesterolemia, and heart disease [3–7]. 

Liver injury is a frequent and multivariate phenomenon which can have dangerous and even fatal consequences. Liver damage involves in most cases oxidative stress and is characterized by a progressive evolution from steatosis to chronic hepatitis, fibrosis, cirrhosis, and hepatocellular carcinoma [8]. For this reason, the search for hepatoprotectors has been intense during the last decades. Hepatoprotection has been attributed to many substances or natural extracts. This includes several aqueous extracts of mushrooms, as for example, *Ganoderma lucidum* [9] and *Ganoderma tsugae* [10]. *A. blazei* extracts (1500 mg/day) have been reported to normalize the liver functions (aminotransferases) of patients with hepatitis B when given at doses of 1500 mg/day [11]. It has also been reported that the mushroom is able to diminish the hepatotoxic effects induced by diethylnitrosamine in rats [12]. More recently it has been shown that a hydroalcoholic extract of *A. blazei* exerts several purinergic effects in the rat liver [13]. These effects were attributed to the various nucleotides and nucleosides which have been demonstrated to exist in *A. blazei* extracts. 

Taken as a whole the investigations that have so far been done with *A. blazei* do not allow much more than preliminary conclusions about the possibilities of the mushroom as a general protective or therapeutic agent [6]. For this reason we decided to start a systematic investigation of the possible protective action of *A. blazei.* The experimental model that was chosen was the tissue injury induced in rats by paracetamol [14]. The latter, a commonly used analgesic, effectively reduces fever and mild to moderate pain and is considered to be safe at therapeutic doses. Paracetamol overdose, however, causes severe hepatotoxicity that leads to liver failure in both humans and experimental animals [15–17]. In all previous studies in which the influence of edible mushroom extracts on paracetamol injury was investigated, the measurements were concentrated on the liver in which histology, enzyme markers and oxidative stress markers were assayed [14]. In the present study attempts were made to extend these measurements to other parameters and other tissues in addition to the liver. For this purpose the actions of paracetamol and an *A. blazei* extract on several parameters in the brain tissue were also investigated. This tissue was chosen taking into account the high content in nucleosides of the *A. blazei* extract [13] and because there are reports about beneficial effects of these compounds on the neural tissue [18]. Moreover, in the liver, the investigations were expanded in order to encompass also functional parameters such as gluconeogenesis, oxygen consumption, and other parameters related to lipid metabolism. The results are hoped to contribute for a better understanding of the action of mushroom extracts on the liver and brain. 

## 2. Materials and Methods

### 2.1. Materials

 Commercial diagnostic kits were used for the plasma analyses. All other chemicals used were in the purest form available commercially. 

### 2.2. Preparation of the *A. blazei* Extract

Fruiting bodies (basidiocarps) of *A. blazei* Murrill were obtained from a local producer in Maringá, PR, Brazil, in Spring 2009. The dried basidiocarps were milled until obtaining a fine powder. The samples (10 g) were extracted by stirring with 100 mL of water (28°C) at 130 rpm for 3 hours and filtered through Whatman paper n°1. The extraction was repeated three times. The filtrates (yield 50%) were lyophilized and stored in freezer until use. 

### 2.3. Animals, Experimental Protocol, and Tissue Preparation

Male albino rats (*Wistar*), weighing 250–300 g, were fed *ad libitum* with a standard laboratory diet (Purina). The rats were maintained in automatically timed light and dark cycles of 12 hours. The present study was approved by the Ethics Committee for the Use of Experimental Animals of the Universidade Estadual de Maringá. 

Rats were distributed randomly into four groups. To the rats of group I (the control group) saline (0.9% NaCl) was administered orally each day during 21 days. Rats of group II were treated orally with the *A. blazei* extract during 21 days; the dosage was 200 mg × (kg body weight)^−1^ × day^−1^. Group III was the hepatotoxicity group to which paracetamol (Tylenol) was given orally as a single dose of 2 g × (kg body weight)^−1^. Group IV was treated with the *A. blazei* extract in the same way as group II during 21 days; at this time paracetamol was given as a single dose of 2 g × (kg body weight)^−1^. Animals were sacrificed 48 h after the single dose of paracetamol or at the corresponding times for those that did not receive paracetamol by intraperitoneal injection of sodium thiopental (50 mg/body weight). Blood samples were collected and plasma was separated by centrifugation at 2500 rpm for 15 minutes for the hepatic marker enzyme assays. Livers, and brains were excised, weighed, frozen in liquid nitrogen and stored until use. 

For the analyses the frozen livers and brains were weighed and homogenized in 10 volumes of 0.1 M potassium phosphate buffer (pH 7.4). This homogenate was used for determining the oxidative stress parameters lipid peroxidation (TBARS), reduced glutathione (GSH), and protein reduced thiol groups. For the determination of enzymatic activities (CAT, SOD, GR, and GPx) and reactive oxygen species (ROS), the homogenate was centrifuged at 10000 g for 15 min and the resulting supernatants were used for the assays. Protein concentration was measured with the Folin-Ciocalteu reagent, using bovine serum albumin as a standard [19]. 

### 2.4. Plasma Hepatic Markers

Plasma samples were assayed for the hepatic marker enzymes aspartate aminotransferase (AST), alanine aminotransferase (ALT), alkaline phosphatase (ALP), and lactate dehydrogenase (LDH). The results were expressed in international units (IU) per liter. Total and conjugated bilirubin and albumin were also assayed. In all cases commercial diagnostic kits were used. 

### 2.5. Plasma Paracetamol

Plasma paracetamol was evaluated in rats from groups III and IV. The rats were anesthetized with sodium thiopental (50 mg/kg) and blood was collected from the cava vein (5 mL). After collection blood was centrifuged for 4000 rpm during 10 minutes at 4°C. The supernatant was used for paracetamol extraction [20, 21]. The latter was done by adding 6 volumes of ethylacetate. This addition was followed by vigorous shaking during two minutes. An aliquot of 1 mL from the organic phase was used for the spectrophotometric paracetamol determination at 290 nm (*ε* = 0.54 mM^−1^ cm^−1^) [20]. A plasma extract from rats of group I was used as the blank. 

### 2.6. Oxidative Stress Parameters

Lipid peroxidation was evaluated by means of the TBARS assay (thiobarbituric acid reactive substances) [22, 23]. The amount of lipoperoxides was calculated from the standard curve prepared with 1,1′,3,3′tetraethoxy-propane and values were expressed as nmol (mg protein)^−1^. 

A fluorometric assay was used to determine the relative levels of different reactive oxygen species (ROS) [24]. The formation of the oxidized fluorescent derivative 2′,7′-dichlorofluorescein (DCF) was measured with a fluorescence spectrophotometer using excitation and emission wavelengths at 504 and 529 nm, respectively. The results were expressed as nmol of DCF formed per mg of protein (nmol/mg protein).

Reduced glutathione (GSH) levels were measured spectrofluorimetrically (excitation at 350 nm and emission at 420 nm) by means of the o-phthalaldehyde assay as described previously [25]. The results were expressed as nmol of GSH per mg of protein (nmol/mg protein).

The levels of protein thiol groups were determined using 5,5′-dithiobis 2-nitrobenzoic acid (DTNB) [26, 27]. The reaction product was measured spectrophotometrically at 412 nm and the molar extinction coefficient of 1.36 × 10^4^ M^−1^ cm^−1^ was used to express the results as nmol (mg protein)^−1^.

### 2.7. Antioxidant Enzymes

The catalase (CAT) activity was estimated by measuring the change in absorbance at 240 nm using H_2_O_2_ as substrate and expressed as *μ*mol min^−1^ (mg protein)^−1^ [28]. The glutathione reductase (GR) activity was estimated by measuring the change in absorbance at 340 nm using NADPH and GSSG as substrates and expressed as nmol min^−1^ (mg protein)^−1^ [29]. The superoxide dismutase (SOD) activity was estimated by its capacity of inhibiting the pyrogallol autooxidation in alkaline medium. The latter was measured at 420 nm [30]. One SOD unit (U) was considered the quantity of enzyme that was able to promote 50% inhibition and the results were expressed as U (mg protein)^−1^. The glutathione peroxidase (GPx) activity was estimated by measuring the change in absorbance at 340 nm due to NADPH consumption in the presence of H_2_O_2_, GSH, and glutathione reductase and expressed as nmol min^−1^ (mg protein)^−1^ [31, 32].

### 2.8. Liver Perfusion

Hemoglobin-free, non-recirculating perfusion was done according to the technique described elsewhere [33, 34]. For the surgical procedure, the rats were anesthetized by intraperitoneal injection of sodium thiopental (50 mg/kg). After cannulation of the portal and cava veins the liver was positioned in a plexiglass chamber. The constant flow was provided by a peristaltic pump (Minipuls 3, Gilson, France) and was adjusted between 30 and 34 mL/min, depending on the liver weight. The perfusion fluid was Krebs/Henseleit-bicarbonate buffer (pH 7.4) containing 25 mg% bovine-serum albumin, saturated with a mixture of oxygen and carbon dioxide (95 : 5) by means of a membrane oxygenator with simultaneous temperature adjustment (37°C). In the effluent perfusion fluid the following compounds were assayed by means of standard enzymatic procedures: glucose, lactate, pyruvate, ammonia, and urea [29]. The oxygen concentration in the outflowing perfusate was monitored continuously, employing a teflon-shielded platinum electrode adequately positioned in a plexiglass chamber at the exit of the perfusate [34]. Metabolic rates were calculated from input-output differences and the total flow rates and were referred to the wet weight of the liver. 

### 2.9. Hepatic Glycogen and Lipids

The hepatic glycogen was determined enzymatically after freeze-clamping freshly isolated livers from anesthetized fed rats (sodium thiopental, 50 mg/kg) [29]. Portions of approximately 2 g of the freeze-clamped tissue were extracted with 10 mL of 0.6 M perchloric acid. Glycogen in the extract was hydrolyzed using amyloglucosidase. The reaction mixture contained 0.1 M potassium hydrogen carbonate, 0.135 M acetate buffer (pH 4.8), 0.75 units of amyloglucosidase, and 200 *μ*L of the extract. After 2 hours incubation at 40°C the reaction was stopped by adding 500 *μ*L of 0.6 M perchloric acid. The mixture was centrifuged and neutralized with potassium hydrogen carbonate and free glucose was determined with a commercial kit. The results were expressed as *μ*mol glucosyl units per gram liver.

Total lipids were extracted from freshly isolated livers and determined gravimetrically [35]. Portions of approximately 5 g tissue were freeze-clamped with liquid nitrogen and subjected to the successive extraction procedures which culminated with a chloroformic lipidic phase [35]. This phase was transferred to a previously weighed beaker and placed in a stove at 45°C for approximately 3 hours. After chloroform evaporation the beaker was weighed and the results expressed as percentage of total lipids per gram liver. For the determination of triacylglycerols, total cholesterol and high-density lipoprotein cholesterol (HDL), the total lipids were dissolved in 1.0 mL chloroform plus 2.0 mL isopropanol, freeze-dried, and stored in freezer. Determinations of triacylglycerols, total cholesterol, and HDL were made using commercial colorimetric-enzymatic kits. The results were expressed as *μ*g per mg total lipids.

### 2.10. Liver Histology

Samples of the left liver lobe (*n* = 5) were fixed in Bouin for 24 h and transferred to 70% ethanol. After dehydration with solutions of successively higher ethanol proportions (80%, 90%, and 100%) and additional 12 hours in xylol the samples were embedded in parafin. These preparations were cut into 7 *μ*m thick semiserial sections and submitted to hematoxylin-eosin staining. The sections were analyzed under microscope. Histological damage was scored into four damage levels [36]: absent (0), mild (+), moderate (++), and severe (+++). Besides this qualitative analysis, the liver degeneration was evaluated using a high resolution digital camera coupled to a microscope. An appropriate software was used to estimate the percentage of liver degeneration. 

### 2.11. Statistical Analysis

All analyses were performed in triplicate. The data were expressed as means ± standard errors of the man (SEM). One-way analysis of variance (ANOVA) with post-hoc Student-Newman-Keuls testing was done; *P* < 0.05 was adopted as the criterion of significance. 

## 3. Results

### 3.1. Plasma Paracetamol Levels

**Figure 1:**
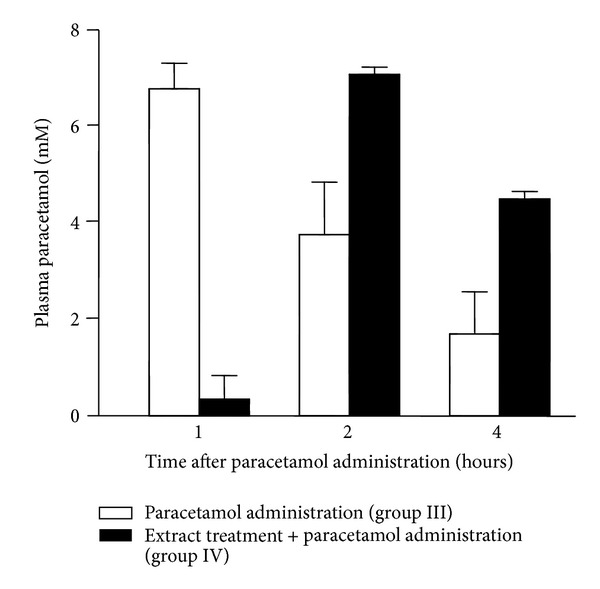
Plasma paracetamol levels at various times after oral administration of 2 g × (kg body weight)^−1^. The four experimental groups (III and IV) are identified on the top. Experimental details can be found in Section 2. Data are means plus mean standard errors of three independent determinations.

The plasma paracetamol levels were measured in rats of groups III and IV during the first 4 hours after administration. The results are shown in Figure 1. In rats of group III (only paracetamol administration) the plasma paracetamol levels were as high as 6.82 mM at one hour after administration, but they declined progressively during the next hours. In the rats of group IV (*A. blazei* pretreatment and paracetamol injury), the highest paracetamol level (7.11 mM) was found two hours after administration. A declining tendency of the plasma levels of paracetamol was also apparent for group IV, even though the decline was clearly delayed with respect to group III. 

### 3.2. Histopathological Evaluations

**Figure 2:**
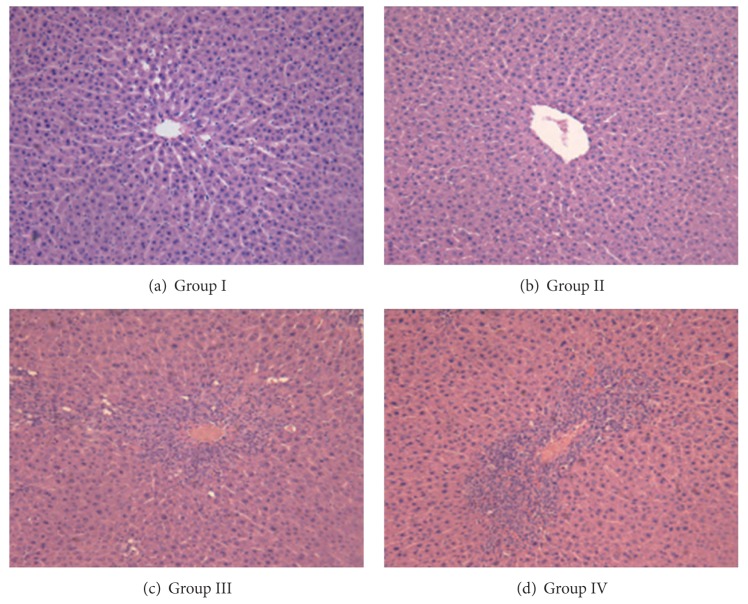
Photomicrographs of rat liver sections stained with hematoxylin and eosin showing the central vein (200x). The four experimental groups (I, II, III, and IV) are identified. Legends for the experimental groups: I, control; II, *A. blazei* pretreatment; III, paracetamol injury; IV, *A. blazei* pretreatment + paracetamol injury.

Figure 2 shows representative photomicrographs of liver sections stained with hematoxylin and eosin.  Table 1 presents the results of the morphological analyses in addition to the liver weights. Groups I and II present the standard histological organization. The sections presented well defined capsules and septa of connective tissue, portal tracts, and central veins with the polyhedral shaped hepatic cells arranged in the form of cords between the sinusoids. The other two groups, III and IV, showed a damaged liver architecture (twisted cords of hepatocytes and veins, disorganized parenchyma), but especially inflammatory infiltrates around the central veins, with the presence of neutrophils and monocytes. In some specimens inflammatory foci around the hepatic paren-chyma were visible. All these observations reflect in the liver damage scores listed in Table 1 and in the estimate of liver degeneration. Both parameters were not significantly modified by the *A. blazei* pretreatment. The fresh liver weight presented a tendency to increase in consequence of the paracetamol injury, but statistical significance at the 5% level was lacking. 

### 3.3. Plasma Hepatic Markers

The levels of enzyme markers that are generally considered as indicative of liver damage are listed in Table 2. The *A. blazei* extract treatment did not produce significant alterations in the parameters listed in Table 2. The paracetamol injury, however, increased all the enzymes that were measured: AST, ALT, LDH, and ALP. It also increased the conjugated bilirubin levels with a nonsignificant tendency for increasing also total bilirubin. The albumin concentration was not affected by the paracetamol injury. The *A. blazei* extract pretreatment reduced considerably the release of hepatic enzymes induced by paracetamol. The lactate dehydrogenase levels, particularly, were close to normal in group IV. The *A. blazei* pretreatment also reduced the appearance of conjugated bilirubin which had been greatly increased by the paracetamol injury. 

### 3.4. Oxidative Stress Assays

Table 3 lists the results of several oxidative stress markers in both brain and liver. Paracetamol administration had a marked influence on the levels of thiobarbituric acid reactive substances (TBARS). In both tissues the TBARS were doubled or nearly so upon paracetamol injury. Remarkably, also in both tissues, the *A. blazei* extract pretreatment effectively prevented the extra TBARS formation induced by paracetamol. The GSH levels suffered a small reduction upon paracetamol injury, an effect that was also prevented by the *A. blazei* extract pretreatment. For the other parameters that were measured, ROS and protein thiol groups, statistical significance was lacking for the eventually small modifications caused by paracetamol or *A. blazei* pretreatment. It must equally be mentioned that the *A. blazei* pretreatment alone was without effect on the variables listed in Table 3. 

### 3.5. Antioxidant Enzyme Assays

*A. blazei* pretreatment did not affect the activites of the four enzymes that were measured in the present work in both brain and liver, as shown in Table 4. Paracetamol injury, however, diminished significantly the activities of catalase and superoxide dismutase in both liver and brain; glutathione reductase and glutathione peroxidase were diminished only in the brain, though to a relatively small extent. The diminution of the catalase activity by paracetamol was quite pronounced: 55% in the liver and 25% in the brain. It is noteworthy that the catalase activity in the brain performs only a small fraction of the activity in the liver (1.5%). In *A. blazei* pretreated rats paracetamol was considerably less effective in promoting the reduction of the enzymatic activities. This is especially true for catalase, whose activities were close to normal in paracetamol injured + *A. blazei* pretreated rats. 

### 3.6. Liver Metabolism

**Figure 3:**
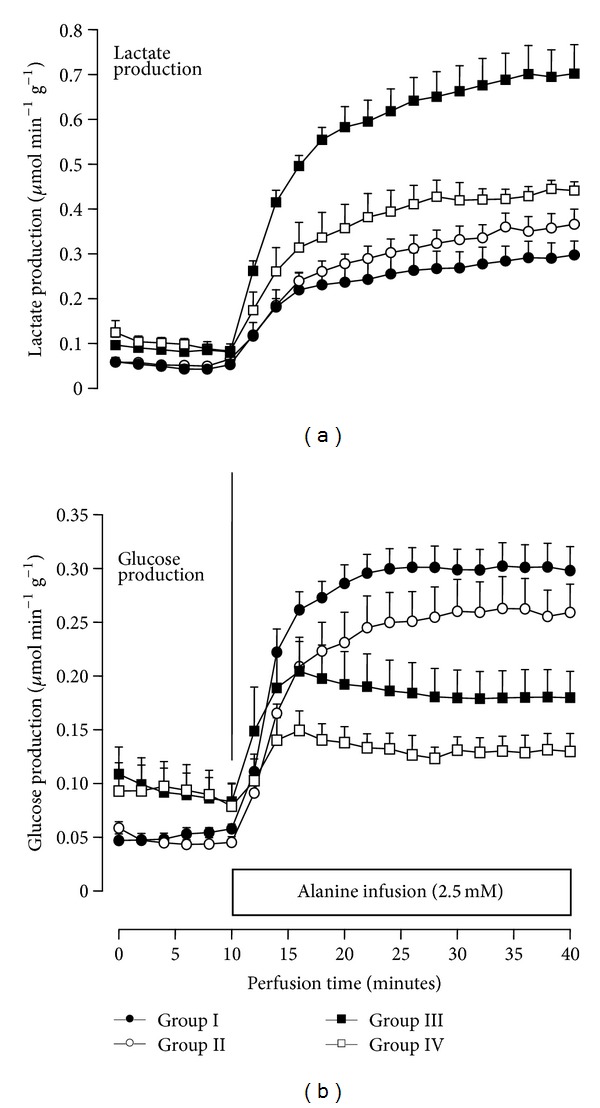
Time course of lactate (a) and glucose (b) production from alanine in the perfused liver from rats of four experimental groups. Livers from fasted rats were perfused as described in Section 2. Alanine was infused during 30 minutes (10 to 40 minutes perfusion time). Samples of the effluent perfusion fluid were collected for the enzymatic metabolite assay. Group I, control; group II, *A. blazei* pretreatment; group III, paracetamol injury; group IV, *A. blazei* pretreatment + paracetamol injury.

Liver metabolism was measured in order to verify if paracetamol injury also affects metabolic routes in the liver and if *A. blazei* pretreatment has some protective action. Alanine metabolism was chosen because the use of this substrate allows measuring simultaneously a biosynthetic route (gluconeogenesis) and evaluating both carbon and nitrogen metabolism. Food was withdrawn from the rats 18 h before the experiments in order to minimize the interference of glycogen catabolism on gluconeogenesis [37].  Figure 3 shows the time courses of glucose and lactate production in response to alanine infusion in perfused rat livers belonging to the four experimental groups used in the present work. The variables shown in Figure 3 were those more strongly affected by both paracetamol and *A. blazei*.  Figure 3 also illustrates the experimental protocol that was employed. Glucose release, lactate production, and several additional parameters were measured before (basal rates) and during alanine infusion. Livers from fasted rats when perfused with a substrate-free medium respire mainly at the expense of endogenous fatty acids [33]. Glycogen content of these livers is quite small, a situation that also reflects in proportionally small rates of metabolite release [37, 38]. Even so it is noteworthy to mention that these residual rates of glucose release and lactate production were more pronounced in livers from paracetamol injured rats, namely, groups III and IV. The basal rates of glucose release in groups III and IV were 0.087 ± 0.011 and 0.094 ± 0.014 *μ*mol min^−1^ g^−1^, respectively. These rates differ from those in groups I and II (*P* < 0.05) which were equal to 0.048 ± 0.005 and 0.047 ± 0.005 *μ*mol min^−1^ g^−1^, respectively. The same was found for lactate production: 0.086 ± 0.011 and 0.10 ± 0.02 *μ*mol min^−1^ g^−1^, respectively, for groups III and IV, but 0.050 ± 0.002 and 0.046 ± 0.005 *μ*mol min^−1^ g^−1^, respectively, for groups I and II (*P* < 0.05). These observations mean a more accentuated glycogen degradation in livers from paracetamol injured rats, a phenomenon that was not affected by the *A. blazei* pretreatment. Upon alanine infusion (Figure 3) both lactate and glucose production increased due to alanine transformation. The excess glucose production is now the consequence of gluconeogenesis and it is quite apparent that the paracetamol injury had a strong negative effect (Figure 3(b)). *A. blazei* pretreatment alone also tended to be inhibitory, an effect that was further enhanced in rats with paracetamol injury. Lactate production derived from alanine transformation, on the other hand, was greatly increased by the paracetamol injury (Figure 3(a)). The *A. blazei* pretreatment was able to prevent this action in part.

Table 5 complements the analysis of the perfusion experiments in two ways: (a) the metabolic rates are given in terms of the increments caused by alanine; that is, they represent only alanine metabolism; (b) all other variables that were measured are represented in addition to glucose and lactate production. The basal rates of the variables other than glucose and lactate production were not different in the four experimental groups (not shown). They were generally very small with the exception of oxygen uptake which was always around 2 *μ*mol min^−1^ g^−1^. Table 5 reveals that, in addition to lactate production, the paracetamol injury also increased pyruvate and ammonia productions from alanine. Urea production was not affected by paracetamol, but it was diminished by the *A. blazei* pretreatment. The oxygen uptake increment caused by alanine was not significantly affected by paracetamol, but there was a tendency toward diminution in livers from *A. blazei* pretreated rats. With the exception of the alanine derived lactate production the *A. blazei* pretreatment did not prevent the metabolic alterations caused by paracetamol. 

### 3.7. Hepatic Levels of Glycogen and Lipids

The hepatic glycogen and lipid contents are listed in Table 6. Livers from fed rats were utilized. As expected from previous studies [39], the paracetamol injury diminished the glycogen levels (group III). *A. blazei* pretreatment alone was without effect on the glycogen levels, and it also failed to prevent the depleting action of paracetamol in a significant way (group IV).

The paracetamol injury did not affect the total fat content of the liver nor did *A. blazei* pretreatment have any action on this variable. The same can be said about the hepatic triglyceride contents. The hepatic cholesterol content, how-ever, which comprises only a small fraction of the total lipid content, was considerably increased in rats injured by paracetamol (+213%). This action was effectively prevented by the *A. blazei* pretreatment. The HDL-cholesterol levels were also increased by paracetamol (+174%). Singularly, the *A. blazei* pretreatment also prevented this increase in spite of the fact that the *A. blazei* pretreatment alone also caused a substantial increase in the HDL-cholesterol levels. 

## 4. Discussion

### 4.1. General Aspects

In the present work the action of an *A. blazei* extract pretreatment on the paracetamol injury was examined not only in terms of the classical indicators, such as levels of hepatic enzymes in the plasma, levels of antioxidant enzymes in the liver and brain tissues, and oxidative stress indicators [14], but also in terms of some functional and metabolic parameters. The classical experimental protocol of the paracetamol injury model was followed in which several indicators are evaluated at 48 hours after oral paracetamol administration [14]. The *A. blazei* pretreatment was able to modify several aspects of the responses of the rat to paracetamol. One of these aspects was the appearance of paracetamol in the plasma, for which a shift of approximately one hour in the time for maximal concentration was found. It is difficult to infer the causes of this phenomenon from the available data. Important for the interpretation of the results, however, is the question whether the shift in the time for maximal concentration could be responsible for the effects of the *A. blazei* extract. The peak concentrations in nontreated and *A. blazei* pretreated rats were the same and the decreases in the plasma paracetamol concentration during the first hour after the time at which the peak concentrations were observed were also approximately the same. This leads to the conclusion that the *A. blazei* pretreatment did not affect the hepatic biotransformation of paracetamol, which is central to its toxicity. In principle at least, it can be hypothesized that the *A. blazei* extract might have delayed intestinal absorption of paracetamol, but this is a question that remains to be elucidated by future work. On the other hand, one hour delay represents a minimal fraction of the 48 hours period after which the injury parameters were measured. It is, thus, unlikely that the phenomenon could have a significant influence on the values of the various parameters that were measured. 

 In the following sections, the action of *A. blazei* will be discussed in terms of both the classical indicators for tissue damage as well as in terms of the functional and metabolic parameters.

### 4.2. Classical Parameters of Hepatoprotection

The most pronounced oxidative damage caused by paracetamol, which also plays a likely role in membrane damage, seems to be lipid peroxidation, which is revealed by the high TBARS levels found in both liver and brain. The aqueous extract of *A. blazei* was able to maintain normal levels of lipid peroxidation (TBARS) in both tissues. The phenomenon can, in principle at least, be attributed to the free-radical scavenging ability of the *A. blazei* extract [40]. This free-radical scavenging ability can be attributed largely to its high content in phenolic compounds because the three phenolics that have been identified in *A. blazei*, gallic acid, syringic acid, and pyrogallol have been also demonstrated to possess high antioxidant activities [41]. Furthermore, their pronounced hydrophilic character makes it highly probable that they are effectively present in the aqueous *A. blazei* extract used in the present study. Phenolic antioxidants act as scavengers of radicals, and sometimes as metal chelators, acting both in the initiation step and the propagation of oxidation. Intermediate products formed by the action of these antioxidants are relatively stable due to resonance of the aromatic ring by these substances [42]. Antioxidant action can also be exerted by polysaccharides, whose presence in the *A. blazei* extract is well documented [43, 44]. In line with this proposition it must be mentioned that the hepatoprotective action of partially purified fungal polysaccharides has been recently demonstrated [45, 46]. Further components of *A. blazei* with antioxidant activity might also be oligopeptides. In fact an oligopeptide from *A. blazei*, rich in Pro, Lys, and Phe and possessing antioxidant activity has been described [47].

The diminution of the GSH levels in both liver and brain upon paracetamol injection was not very pronounced, at least not at 48 hours after the injury. Even so the effect of the *A. blazei* treatment was in the direction of normalization in both liver and brain. In addition to the effects on the TBARS levels, another prominent effect of the *A. blazei* extract pretreatment against the paracetamol injury was that on the membrane integrity. This was evidenced by the hepatic enzyme levels in the plasma (AST, ALT, LDH, and ALP), whose increases due to paracetamol injury were in all cases markedly prevented by the *A. blazei* treatment. 

In relation to the activities of antioxidant enzymes CAT, SOD, GPx, and GR, the paracetamol injury diminished only the first two in the liver. In the brain paracetamol diminished all these activities, though to lesser extents. Interpretation of the results is not easy because it is difficult to correlate the pronounced diminution of the hepatic CAT levels caused by the paracetamol injury with the absence of a significant increase in the hepatic ROS levels. The *A. blazei* pretreatment partially prevented the consequences of paracetamol injury, most notably for the hepatic CAT levels and the brain SOD and GR levels, which were preserved close to normal. It is possible that this phenomenon was at least partly caused by adenosine or other purinergic agents which have been demonstrated to be important components of *A. blazei* [13]. Nucleosides and nucleotides are purinergic agents and purinergic effects of an *A. blazei* extract have been recently demonstrated to occur in the rat liver [13]. Adenosine, but possibly also other activators of A_1_ purinergic receptors, confers cytoprotection in the cardiovascular and central nervous systems by activating cell surface adenosine receptors [48, 49]. Activation of these receptors, in turn, is postulated to activate antioxidant enzymes via protein kinase C phosphorylation of the enzymes or of intermediates that promote activation [48]. 

It should be remarked that in respect to the parameters discussed above, the action of the *A. blazei* extract pretreatment on the paracetamol injury is similar to that reported for the injury induced by carbon tetrachloride [50]. The same action was observed in patients with hepatitis B [11]. Also, extracts of other mushroom species have been shown to act similarly to the *A. blazei* extract on paracetamol injury, namely, *Lentinula edodes*, *Grifola frondosa* and *Tricholoma labayense* [14]. It must be remarked that other species, namely, *Volvariella volvacea, Flammulina velutipes, Auricularia auricular, *and* Tremella fuciformis, *failed to exert significant hepatoprotective actions [13] so that the hepatoprotective ability is not a universal characteristic among mushrooms. 

### 4.3. Functional and Metabolic Parameters

Gluconeogenesis impairment was one of the most important consequences of paracetamol injury. The phenomenon reported in the present work bears probably little or no relation to the same phenomenon reported earlier [20] in the isolated perfused rat liver under constant paracetamol infusion. Under these conditions paracetamol inhibits gluconeogenesis by virtue of its inhibitory action on oxidative phosphorylation, which is a reversible effect. As long as paracetamol is present in the circulation it will obviously be exerting this action, but in the present work the livers were perfused 48 hours after paracetamol administration and the small amount of drug still present was rapidly washed out into the perfusion fluid. Consequently, the metabolic effects of paracetamol, including gluconeogenesis diminution, were most likely the consequence of the injury induced by the drug. In this respect the signs of inflammation that were observed, especially the presence of neutrophils and monocytes in addition to the inflammatory foci around the hepatic parenchyma, are highly significant. Inflammatory states are frequently, if not always, associated with decreased rates of gluconeogenesis. For example, diminished gluconeogenesis was found in livers from arthritic rats [51], in livers from rats poisoned with the venom of *Loxosceles intermedia* [52], and also in livers from rats injected with the inflammatory cytokines [53]. It seems thus reasonable to conclude that impairment of gluconeogenesis by paracetamol is closely associated with the inflammatory state. The *A. blazei* pretreatment was unable to prevent both. The pretreatment was also not effective in preventing the increased ammonia production induced by paracetamol, a parameter that indicates some kind of impairment of ammonia detoxification. The only normalizing effect of *A. blazei* on the metabolic effects of paracetamol was that on lactate production. These observations, in addition to the finding that the extract itself caused a diminished urea production, indicates some degree of interference with the hepatic metabolism. This interference must be the result of medium- and long-term effects because the components of the extract able to exert short-term effects as well, such as nucleosides and nucleotides [13], are no longer present in the isolated perfused liver. It should be added that it is unlikely that the gluconeogenesis impairment caused by paracetamol is a consequence of the loss of enzymes such as alanine aminotransferase. Lactate and pyruvate productions were increased by the paracetamol injury and alanine transformation into these products occurs necessarily via alanine amino-transferase. Furthermore, the *A. blazei* treatment reduced significantly the paracetamol-induced release of ALT and other enzymes, without preventing the negative effect on gluconeogenesis.

Pretreatment with the *A. blazei* aqueous extract had also little influence on the paracetamol diminished hepatic glycogen levels. The latter is a well-known consequence of paracetamol overdosing [39], which is likely to be related to the gluconeogenesis impairment if one considers that a significant fraction of the hepatic glycogen synthesis depends on this metabolic pathway [54]. Singularly, however, the *A. blazei* pretreatment was able to prevent the increased levels of hepatic cholesterol induced by paracetamol. The data available so far do not allow any mechanistic interpretation for this phenomenon, but this observation indicates again that the *A. blazei* extract is able to exert metabolic effects. It is worth to mention that the hydroalcoholic extract of the flowers of *Calotropis procera* was also found able to normalize the cholesterol levels in rats of paracetamol injured rats [55]. 

## 5. Conclusions

If one considers only the classical indicators for tissue damage, the results that were obtained in the present study can be regarded as a positive indication that the *A. blazei* extract is able to provide a reasonable degree of protection against the paracetamol injury in both the hepatic and brain tissues. With respect to the functional and metabolic parameters of the liver, however, the extract provided little or no protection against most alterations caused by paracetamol. This includes microscopic morphological characteristics and the especially important functional parameter gluconeogenesis. This and other functional parameters, however, are usually not measured when hepatoprotection is examined. Considering our results and the long list of extracts and substances that are said to have hepatoprotective effects, however, it would be useful to incorporate evaluations of functional parameters into the experimental protocols of studies aiming to attribute or refute effective hepatoprotective actions.

## Figures and Tables

**Table 1:** Weight and morphological characteristics of rat livers from the four experimental groups used in the present study. Means and observations were derived from 5 animals in each group. Histological damage was scored into four damage levels: absent (0), mild (+), moderate (++), and severe (+++). The software Image-Pro Plus 4.5 was used to estimate the percentage of liver degeneration. For additional experimental details and a more complete description of the experimental groups see Section 2.

Groups	Liver weights	Morphological aspects	Liver damage scores				Estimates of liver degeneration
			0	+	++	+++	
I, control	9.02 ± 0.69	Uniformly red and soft consistency	5	0	0	0	—
II, *A. blazei* pretreatment	9.16 ± 1.17	Uniformly red and soft consistency	5	0	0	0	—
III, paracetamol injury	11.50 ± 0.86	Larger, whitish, and with nodules	0	1	3	1	11.78%
IV, *A. blazei* pretreatment + paracetamol injury	10.40 ± 1.61	Larger, whitish, and with nodules	1	0	3	1	9.89%

**Table 2:** Effects of paracetamol injury and *A. blazei* pretreatment on plasma enzyme activities, albumin levels, and bilirubin concentration. For experimental details see Section 2.

Parameters	Groups			
	I, control	II, *A. blazei* pretreatment	III, paracetamol injury	IV, *A. blazei* pretreatment + paracetamol injury
Aspartate aminotransferase (AST, U/L)	74.6 ± 9.3 (*n* = 4)	71.6 ± 4.0 (*n* = 4)	512.9 ± 62.7 (*n* = 4)^a,b,c^	246.6 ± 65.2 (*n* = 4)^d,e^
Alanine aminotransferase (ALT, U/L)	65.3 ± 4.2 (*n* = 5)	63.7 ± 2.6 (*n* = 4)	447.0 ± 52.0 (*n* = 4)^a,b,c^	280.2 ± 64.0 (*n* = 4)^d,e^
Lactate dehydrogenase (LDH, U/L)	248.9 ± 12.5 (*n* = 4)	227.7 ± 38.1 (*n* = 4)	531.0 ± 36.8 (*n* = 5)^a,b,c^	304.4 ± 24.7 (*n* = 4)
Alkaline phosphatase (ALP, U/L)	74.6 ± 14.8 (*n* = 4)	72.6 ± 3.6 (*n* = 4)	174.1 ± 16.2 (*n* = 5)^a,b,c^	123.3 ± 5.6 (*n* = 5)^d,e^
Albumin (g/L)	2.37 ± 0.09 (*n* = 5)	2.21 ± 0.14 (*n* = 5)	2.48 ± 0.20 (*n* = 4)	2.22 ± 0.10 (*n* = 6)
Total bilirubin (mg/dL)	0.53 ± 0.01 (*n* = 4)	0.35 ± 0.01 (*n* = 4)	0.80 ± 0.01 (*n* = 5)^b^	0.64 ± 0.11 (*n* = 6)^e^
Conjugated bilirubin (mg/dL)	0.07 ± 0.01 (*n* = 4)	0.07 ± 0.01 (*n* = 4)	0.24 ± 0.01 (*n* = 4)^a,b,c^	0.17 ± 0.01 (*n* = 6)^d,e^

Significant differences (*P* ≤ 0.05), according to one-way ANOVA followed by Student-Newman-Keuls post-hoc testing, are identified by the superscript letters as follows: ^a^III versus I; ^b^III versus II; ^c^III versus IV; ^d^IV versus I; ^e^IV versus II.

**Table 3:** Effects of paracetamol injury and *A. blazei* pretreatment on oxidative stress indicators in the liver and brain tissues. For experimental details see Section 2.

Parameters	Organ	I, control	II, *A. blazei* pretreatment	III, paracetamol injury	IV, *A. blazei* pretreatment + paracetamol injury
TBARS (nmol mg^−1^)	Liver	1.05 ± 0.19 (*n* = 4)	1.01 ± 0.18 (*n* = 4)	2.08 ± 0.23 (*n* = 3)^a,b,c^	1.08 ± 0.16 (*n* = 4)
	Brain	2.64 ± 0.08 (*n* = 6)	2.73 ± 0.11 (*n* = 6)	3.29 ± 0.21 (*n* = 6)^a,b,c^	2.61 ± 0.18 (*n* = 6)
ROS (nmol mg^−1^)	Liver	1.96 ± 0.38 (*n* = 3)	2.31 ± 0.28 (*n* = 3)	2.48 ± 0.48 (*n* = 3)	2.23 ± 0.47 (*n* = 4)
	Brain	8.31 ± 0.91 (*n* = 5)	8.63 ± 0.66 (*n* = 5)	9.62 ± 0.69 (*n* = 4)	8.61 ± 0.74 (*n* = 4)
GSH (nmol mg^−1^)	Liver	13.66 ± 0.65 (*n* = 4)	13.14 ± 0.71 (*n* = 4)	9.76 ± 0.91 (*n* = 4)^f,g^	12.66 ± 2.02 (*n* = 3)
	Brain	8.82 ± 0.19 (*n* = 6)	8.79 ± 0.61 (*n* = 6)	7.31 ± 0.23 (*n* = 5)^h^	8.28 ± 0.45 (*n* = 8)
Protein thiol groups (nmol mg^−1^)	Liver	115.3 ± 14.0 (*n* = 4)	122.6 ± 14.7 (*n* = 4)	80.7 ± 7.4 (*n* = 4)	101.1 ± 2.0 (*n* = 4)
	Brain	95.2 ± 5.5 (*n* = 5)	95.0 ± 4.8 (*n* = 5)	84.4 ± 3.6 (*n* = 7)	84.4 ± 3.5 (*n* = 6)

Significant differences (*P* ≤ 0.05), according to one-way ANOVA followed by Student-Newman-Keuls post-hoc testing, are identified by the superscript letters as follows: ^a^III versus I; ^b^III versus II; ^c^III versus IV. Significant differences according to Student's *t*-test are given by the codes: ^f^ *P* = 0.013 for III versus I; ^g^ *P* = 0.026 for III versus I; ^h^ *P* < 0.001 for III versus I.

**Table 4:** Effects of paracetamol injury and *A. blazei* pretreatment on antioxidant enzyme activities in the liver and brain tissues. For experimental details see Section 2.

Enzymes	Organ	I, control	II, *A. blazei* pretreatment	III, paracetamol injury	IV, *A. blazei* pretreatment + paracetamol injury
Catalase (*μ*mol min^−1^ mg^−1^)	Liver	1078.8 ± 75.1 (*n* = 4)	942.5 ± 104.1 (*n* = 3)	597.0 ± 68.2 (*n* = 4)^a,b,c^	898.3 ± 33.9 (*n* = 4)
	Brain	15.97 ± 0.79 (*n* = 5)	16.79 ± 1.21 (*n* = 5)	12.00 ± 0.98 (*n* = 6)^a,b^	14.56 ± 0.77 (*n* = 5)

Superoxide dismutase (units mg^−1^)	Liver	4.29 ± 0.14 (*n* = 4)	4.3 ± 0.42 (*n* = 4)	2.97 ± 0.17 (*n* = 4)^a,b^	3.30 ± 0.22 (*n* = 4)^d,e^
	Brain	2.03 ± 0.13 (*n* = 5)	1.97 ± 0.09 (*n* = 5)	1.46 ± 0.09 (*n* = 6)^a,b,c^	1.78 ± 0.10 (*n* = 7)

Glutathione reductase (nmol min^−1^ mg^−1^)	Liver	36.11 ± 3.11 (*n* = 4)	38.38 ± 2.04 (*n* = 4)	46.25 ± 5.85 (*n* = 3)	47.25 ± 1.82 (*n* = 3)
	Brain	14.34 ± 0.73 (*n* = 6)	15.58 ± 0.99 (*n* = 6)	12.76 ± 0.43 (*n* = 8)^b,c^	14.99 ± 0.44 (*n* = 8)

Glutathione peroxidase (nmol min^−1^ mg^−1^)	Liver	107.2 ± 5.7 (*n* = 3)	133.8 ± 9.0 (*n* = 3)	122.3 ± 16.2 (*n* = 3)	139.7 ± 16.1 (*n* = 3)
	Brain	33.0 ± 1.3 (*n* = 5)	31.5 ± 2.1 (*n* = 5)	27.4 ± 0.5 (*n* = 6)^a^	30.8 ± 0.7 (*n* = 7)

Significant differences (*P* ≤ 0.05), according to one-way ANOVA followed by Student-Newman-Keuls post-hoc testing, are identified by the superscript letters as follows: ^a^III versus I; ^b^III versus II; ^c^III versus IV; ^d^IV versus I; ^e^IV versus II.

**Table 5:** Effects of paracetamol injury and *A. blazei* pretreatment on metabolic parameters of perfused rat livers metabolizing exogenously supplied alanine. Livers from fasted rats were perfused according to the experimental protocol illustrated by Figure 2. For additional experimental details and see Section 2.

Metabolic fluxes (*µ*mol min^−1^ g^−1^)	I, control	II, *A. blazei* pretreatment	III, paracetamol injury	IV, *A. blazei* pretreatment + paracetamol injury
Lactate production	0.31 ± 0.03 (*n* = 5)	0.25 ± 0.03 (*n* = 4)	0.62 ± 0.05 (*n* = 5)^a,b,c^	0.35 ± 0.02 (*n* = 3)
Pyruvate production	0.13 ± 0.02 (*n* = 5)	0.082 ± 0.030 (*n* = 4)	0.29 ± 0.05 (*n* = 5)^a,b^	0.20 ± 0.02 (*n* = 3)
Glucose production	0.25 ± 0.02 (*n* = 5)	0.21 ± 0.02 (*n* = 4)	0.092 ± 0.020 (*n* = 5)^a,b^	0.060 ± 0.030 (*n* = 3)^d,e^
Oxygen consumption	0.39 ± 0.06 (*n* = 5)	0.23 ± 0.05 (*n* = 4)	0.32 ± 0.07 (*n* = 5)	0.29 ± 0.03 (*n* = 3)
Ammonia production	0.058 ± 0.010 (*n* = 5)	0.085 ± 0.030 (*n* = 4)	0.20 ± 0.03 (*n* = 5)^a,b^	0.16 ± 0.01 (*n* = 3)^d^
Urea production	0.28 ± 0.01 (*n* = 5)	0.12 ± 0.01 (*n* = 4)^f,g^	0.23 ± 0.03 (*n* = 5)^b^	0.25 ± 0.05 (*n* = 3)

Significant differences (*P* ≤ 0.05), according to one-way ANOVA followed by Student-Newman-Keuls post-hoc testing, are identified by the superscript letters as follows: ^a^III versus I; ^b^III versus II; ^c^III versus IV; ^d^IV versus I;^ e^IV versus II; ^f^II versus I; ^g^II versus IV.

**Table 6:** Effects of paracetamol injury and *A. blazei* pretreatment on hepatic glycogen and lipid levels. Livers from fed rats were utilized. For additional experimental details see Section 2.

Parameters	I, control	II, extract treatment	III, paracetamol injury	IV, *A. blazei* pretreatment + paracetamol injury
Hepatic glycogen (*μ*mol glucosyl units/g liver)	260.4 ± 16.9 (*n* = 5)	273.3 ± 7.4 (*n* = 6)	202.3 ± 4.5 (*n* = 6)^a,b^	223.8 ± 21.5 (*n* = 5)^d^
Total lipids (g/100 g liver)	3.66 ± 0.13 (*n* = 8)	3.62 ± 0.16 (*n* = 8)	3.11 ± 0.17 (*n* = 8)	3.58 ± 0.19 (*n* = 6)
Hepatic triglycerides (mg/g total lipids)	96.61 ± 7.51 (*n* = 8)	107.98 ± 10.66 (*n* = 8)	113.75 ± 4.56 (*n* = 8)	85.22 ± 19.75 (*n* = 6)
Cholesterol (mg/g total lipids)	39.49 ± 2.99 (*n* = 8)	45.31 ± 3.85 (*n* = 4)	84.49 ± 5.03 (*n* = 8)^a,b,c^	47.24 ± 9.09 (*n* = 6)
HDL cholesterol (mg/g total lipids)	1.64 ± 0.12 (*n* = 8)	2.61 ± 0.20 (*n* = 8)^e^	2.90 ± 0.31 (*n* = 8)^a,c^	1.91 ± 0.36 (*n* = 6)

Significant differences (*P* ≤ 0.05), according to one-way ANOVA followed by Student-Newman-Keuls post-hoc testing, are identified by the superscript letters as follows: ^a^III versus I; ^b^III versus II; ^c^III versus IV; ^d^IV versus I; ^e^II versus I.
